# Supplementary material for: Malaria mosquitoes acquire and allocate cattle urine to enhance life history traits
Source: Malar J. 2022 Jun 11;21:180. doi: 10.1186/s12936-022-04179-6 (PMC9188207; doi:10.1186/s12936-022-04179-6)
Supplement: Supplementary file 1 — Additional file 1: Table S1. Species, sex and gonotrophic state of the mosquitoes captured in CDC light traps baited with the synthetic odour blend of 24 h aged cattle urine or heptane control. Table S2. Synthetic compounds used for electrophysiological and behavioural analyses. Fig S1. Blood-fed Anopheles arabiensis display no oviposition preference for the headspace volatile extracts of fresh and aged cattle urine. Letter designations indicate no significant difference from one another (one-way analysis of variance with a Tukey’s post hoc analysis; p > 0.05). Error bars indicate the standard error of the proportion. Fig. S2. Behavioural responses of host-seeking (A) and blood-fed (B) Anopheles arabiensis to the full and subtractive synthetic blends of 24 h aged cattle urine. The removal of single components from the synthetic blend (open circles) differentially and significantly affected the response of the females from both physiological states. Different lowercase letters indicate significant differences as determined by a one-way analysis of variance followed by a Dunnett’s post hoc analysis (p < 0.05). Error bars represent the standard error of proportion. Fig. S3. Cattle urine enhances host decoy trap catches only in the presence of the host cue, heat. Host decoy traps only caught malaria mosquitoes in a deserted pasture between the breeding site and the village in the presence of both heat and cattle urine (fresh or aged), but not either alone. Error bars indicate the standard error of the mean. [file 12936_2022_4179_MOESM1_ESM.pdf]

**Supporting Information**

# Malaria mosquitoes acquire and allocate cattle urine to enhance life history traits

Supporting Tables: 2
Supporting Figures: 3

**Supporting Table 1**

| | | Host-seeking | Blood fed | Semi-gravid | Gravid | Males |
|---|---|---|---|---|---|---|
| CDC light traps | *Anopheles arabiensis* | 466 | 154 | 30 | 71 | 29 |
| | *Anopheles pharoensis* | 141 | 25 | 14 | 23 | 5 |
| | *Culex* spp. | 562 | 100 | 19 | 17 | 9 |
| CDC light traps baited with synthetic blend | *Anopheles arabiensis* | 879 | 363 | 85 | 109 | 37 |
| | *Anopheles pharoensis* | 471 | 123 | 29 | 62 | 26 |
| | *Culex* spp. | 757 | 164 | 22 | 57 | 12 |

**Supporting Table 2.**

| **Compound** | **Compound class** | **CAS No.** | **Purity (%)** | **Supplier** |
|---|---|---|---|---|
| 2-ethyl-1-hexenol | aliphatic alcohol | 104-76-7 | 99 | Sigma-Aldrich |
| decanal* | aliphatic aldehyde | 112-31-2 | 98 | Sigma-Aldrich |
| 3-nonen-2-one | aliphatic ketone | 18402-83-0 | 95 | Sigma-Aldrich |
| 2-cyclohexen-1-one* | cyclic, aliphatic ketone | 930-68-7 | 96 | VWR |
| phenol* | aromatic alcohol | 108-95-2 | 99.5 | Sigma-Aldrich |
| *m*-cresol* | aromatic alcohol | 108-39-4 | 97 | Sigma-Aldrich |
| *p*-cresol* | aromatic alcohol | 106-44-5 | 99 | Sigma-Aldrich |
| 4-ethylphenol | aromatic alcohol | 123-07-9 | 99 | Sigma-Aldrich |
| *S*-(–)-limonene | monoterpenic hydrocarbon | 5989-54-8 | 95 | Sigma-Aldrich |
| linalool* | monoterpenic alcohol | 78-70-6 | 97 | Sigma-Aldrich |

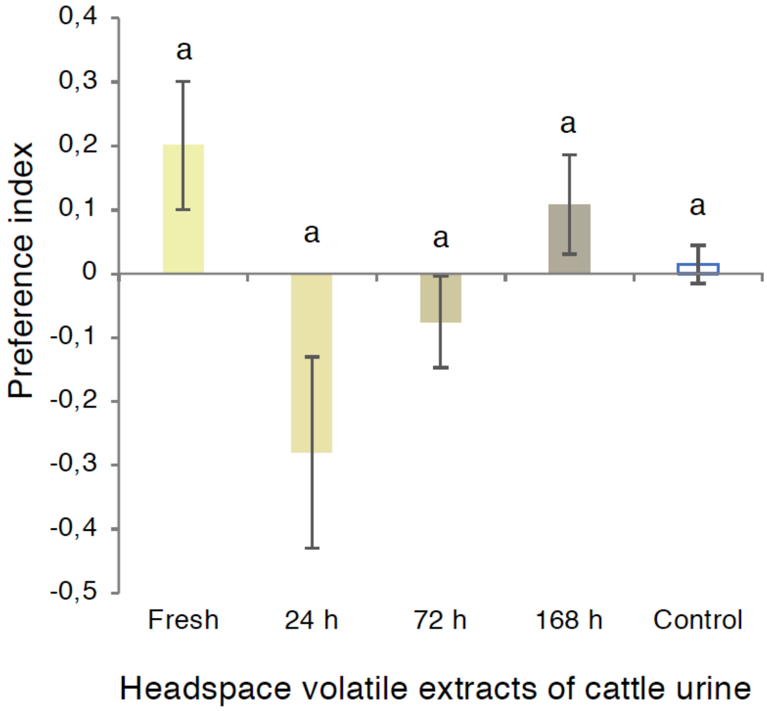


**Supporting Fig 1**

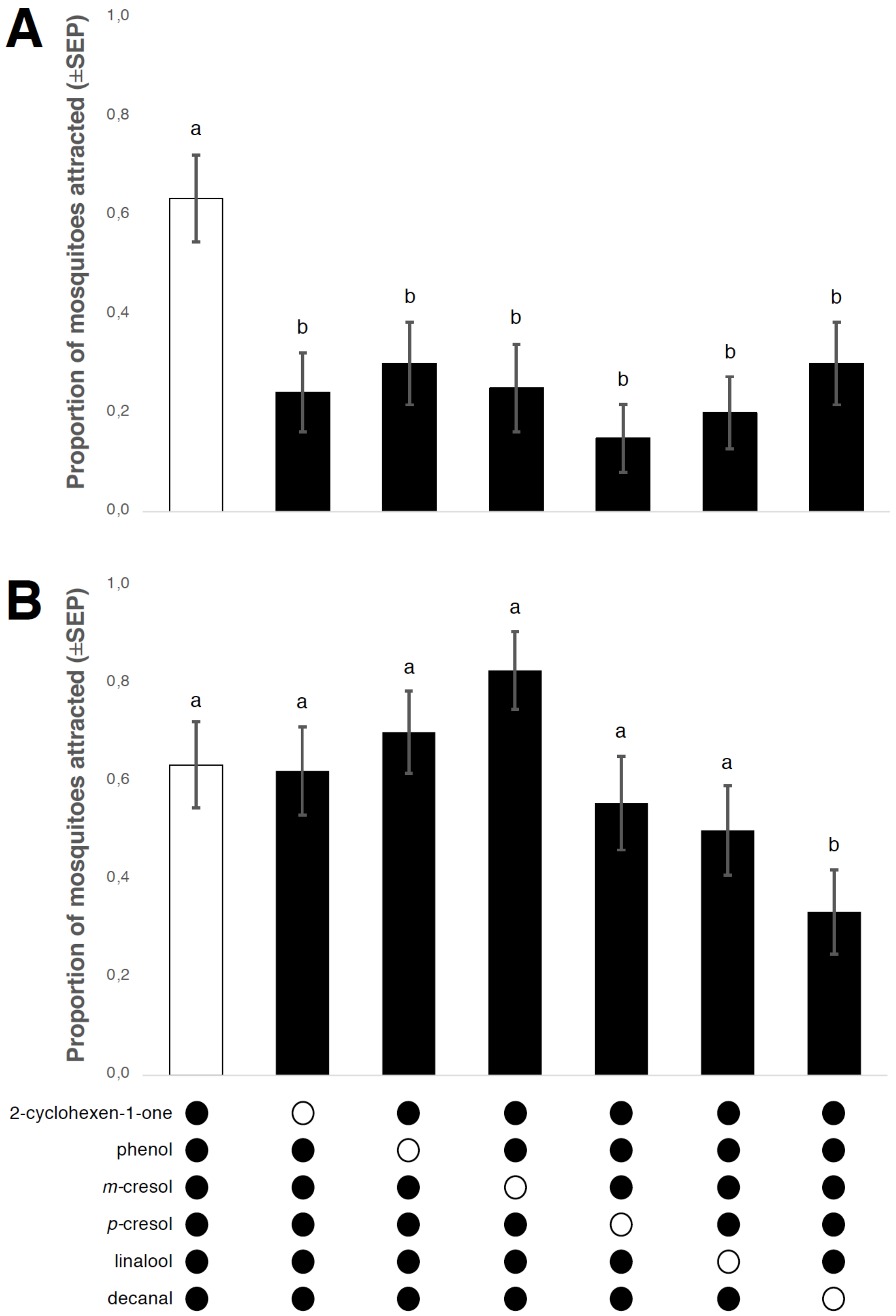


**Supporting Fig 2.**

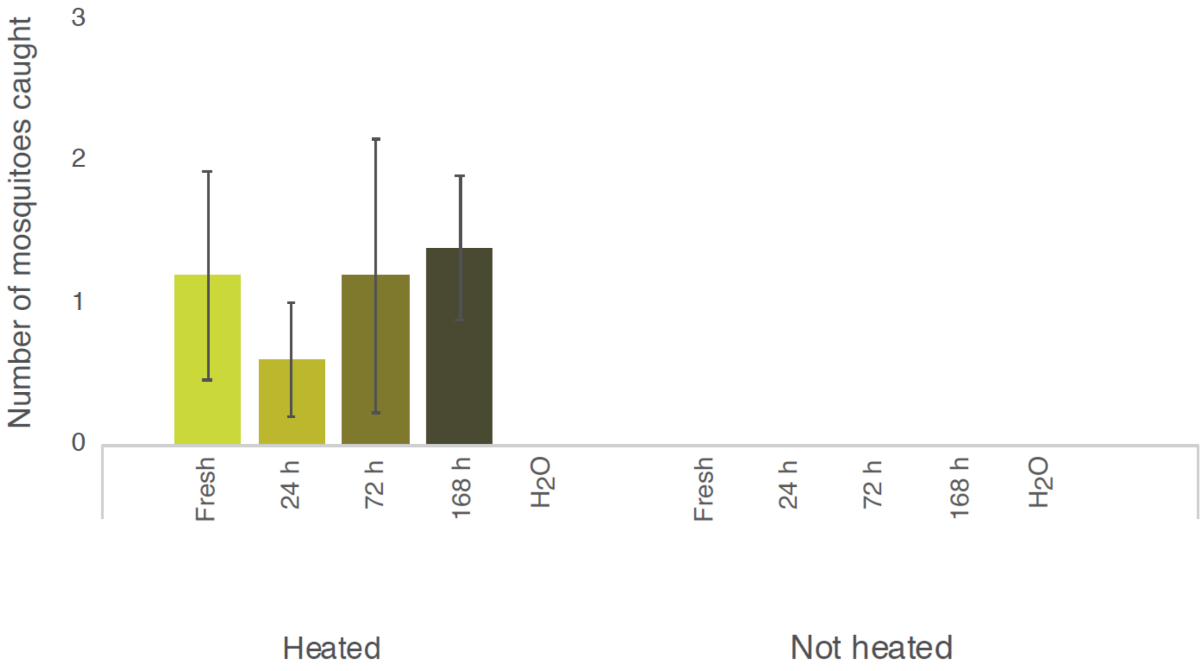


**Supplementary Figure 3. Cattle urine enhances host decoy trap catches only in the presence of the host cue, heat**. Host decoy traps only caught malaria mosquitoes in a deserted pasture between the breeding site and the village in the presence of both heat and cattle urine (fresh or aged), but not either alone. Error bars indicate the standard error of the mean.
